# The impact of air pollutants on the risk of goiter based on a 9-year time series data

**DOI:** 10.3389/fpubh.2025.1663263

**Published:** 2025-09-10

**Authors:** Yanbin Du, Hua Zhou, Yang Chen

**Affiliations:** ^1^College of Mathematics and Statistics, Henan University of Science and Technology, Luoyang, Henan Province, China; ^2^Endocrinology and Metabolism Center, Henan Key Laboratory of Rare Diseases, The First Affiliated Hospital, College of Clinical Medicine of Henan University of Science and Technology, Luoyang, Henan Province, China; ^3^Henan Academy of Innovations in Medical Science, Zhengzhou, Henan Province, China

**Keywords:** air pollution, goiter, generalized additive model, lag effect, environmental health

## Abstract

**Background:**

With the rapid advancement of industrialization and urbanization, air pollution is becoming increasingly serious, posing a huge threat to human health. There is limited literatures to study the relationship between air pollution and thyroid diseases. Therefore, this study aims to investigate the association between air pollutions (PM_2.5_, PM_10_, SO_2_, NO_2_, O_3_, and CO) and thyroid goiter.

**Methods:**

A 9-year time series data was collected from the Luoyang Air Testing Website from 2014 to 2022. A generalized additive model (GAM) based on Poisson regression was established and stratification analysis were used to explore the differences in the population by gender, age, place of residence, and season.

**Results:**

There were 37,630 hospital admissions for goiter in Luoyang from January 1, 2014 to July 30, 2022. Among them, there are 29,571 female (78.58%) and 8,059 male (21.42%); There are different lag effects of air pollutants on the thyroid goiter, and the relative risk (RR) of thyroid goiter showed a non-linear increasing trend with the increase of pollutants concentration on the optimal lag day. A 10 μg/m^3^ increase in PM_2.5_, PM_10_, O_3_, and NO_2_ concentrations (1 mg/m^3^ increase in CO) was associated with a 1.0092%(95%CI: 1.0032–1.015), 1.0044% (95%CI: 1.0008–1.0081), 0.9928%(95%CI: 0.9867–0.9988), 1.0596% (95%CI: 1.0413–1.0783) and 1.624%(95%CI: 1.1347–2.3243) risk of thyroid goiter, respectively. Besides, the effect of SO_2_ on goiter was not statistically significant. The stratified analysis results showed that women, age >45 years old, and urban populations may be more sensitive to pollutants, and people may be more sensitive to pollutants in autumn.

**Conclusions:**

This time-series study suggested that long-term exposure to air pollutions may be associated with an increased risk of thyroid diseases, especially NO_2_ and CO have a greater impact on goiter than PM. These associations were stronger for patients more than 45 years old and during the autumn, especially for women. These findings suggest the importance of reducing air pollutant concentrations and protecting the environment.

## 1 Introduction

The thyroid gland is a very important endocrine organ in the human body, located in the anterior lower part of the neck. The job of the thyroid is the synthesis of thyroid hormones which are responsible for the metabolism in the body. Thyroid lesions can cause significant harm to the human body, goiter is a common clinical sign and manifestation of various thyroid diseases. It's reported by Al-Rekabi and Habban that thyroid tumor rate was 21.6% from patients with goiter (1). According to the latest assessment report released by the International Agency for Research on Cancer (IARC) of the World Health Organization (WHO), there are more than 821,000 new cases of thyroid cancer, and the overall incidence rate ranks seventh in the world (2). According to the report released by China Cancer Center in 2022, the standardized incidence rate of thyroid cancer in China has increased from 1.4/100,000 person years in 1990 to 14.65/100,000 person years in 2016, with a 10 fold increase in incidence rate (3). In 2022, thyroid cancer ranked third in the number of new cancer cases in China, it's urgent need to seek risk factors for thyroid disease.

With the rapid advancement of industrialization and urbanization, air pollution is becoming increasingly serious, posing a huge threat to human health. Especially pollutants such as fine particulate matter (PM_2.5_), ozone (O_3_), and nitrogen dioxide (NO_2_) in the air have been widely studied and confirmed to be closely related to various respiratory diseases (4–6), cardiovascular diseases (7, 8), and cancer (9–11). According to the WHO, the impact of air pollution causes approximately 7 million deaths annually (12). Air pollutants have a wide range of impacts on human health, therefore, reducing air pollution and improving air quality are crucial for maintaining human health.

The only confirmed risk factor for thyroid cancer currently known is ionizing radiation. However, recent several studies have shown that some air pollutants may be related to thyroid dysfunction (13–15) and the increased incidence rate of thyroid diseases (16, 17). Overall, there is limited literature to study the impact of air pollution on thyroid diseases, especially goiter. Therefore, exploring the link between air pollution and goiter is of great significance.

## 2 Materials and methods

### 2.1 Data sources

**Figure 1:**
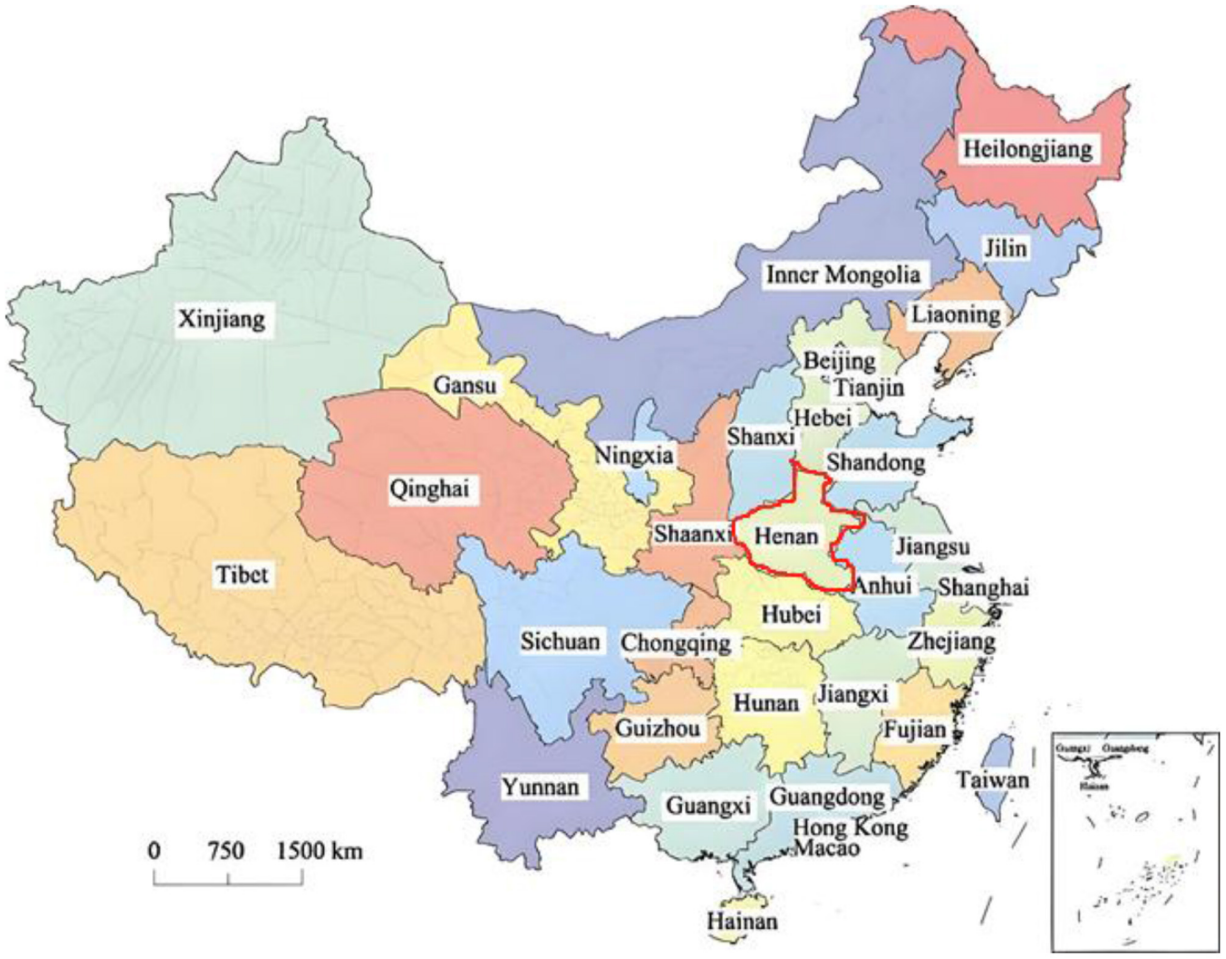
The geographical distribution of Henan Province.

Henan Province is located in the middle and lower reaches of the Yellow River in the middle east of China and the south of the North China Plain, between 31°23′-36°22′N and 110°21′-116°39′E. Luoyang City is located in the western part of Henan Province, it is situated between longitude 112°16′-112°7′ and latitude 34°2′-34°5′, with a length of approximately 179 km from east to west and a width of approximately 168 km from north to south. The detailed geographical location of Henan Province is shown in Figure 1.

We collected daily concentration data of six air pollutants (PM_2.5_, PM_10_, NO_2_, SO_2_, O_3_, and CO) in Luoyang from January 1, 2014 to July 30, 2022 and data from the Luoyang Air Testing Website (https://citydev.gbqyun.com/index/luoyang). There are ten air quality monitoring stations in Luoyang city, the daily concentration of air pollutants was simply an arithmetic mean measure across all the monitoring stations, as in most time-series studies. Daily mean temperature data in Luoyang were from the National Meteorological Information Center (http://data.cma.cn/).

Daily hospital admissions data were collected from the First Affiliated Hospital of Henan University of Science and Technology and The Third People's Hospital, which the two most representative hospitals in Luoyang. The clinical diagnostic criteria for Thyroid diseases from International Classification of Diseases. The patient's goiter was determined by ultrasound, and iodine deficiency patients were excluded. Patients' basic information included gender, age and residence.

### 2.2 GAM model

A generalized additive model (GAM) with a Poisson distribution was adopted to analyze the impact of air pollutions on daily hospital admissions of goiter. The effect of different time lags was examined including eight single-day lags: (i) lag 0, the pre sent day; (ii) lag 1, the previous day; (iii) lag 2, the day before lag 1; (iv) lag 3, the day before lag 2; (v) lag 4, the day before lag 3; (vi) lag 5, the day before lag 4, (vii) lag 6, the day before lag 5, (viii) lag 7, the day before lag 6, and seven moving average exposure lags: (i) lag 01, the 2-day moving average of the present and previous day; (ii) lag 02, the 3-day moving average of the present and previous 2 days; (iii) lag 03, the 4-day moving average of the present and previous 3 days. (iv) lag 04, the 5-day moving average of the present and previous 4 days. (v) lag 05, the 6-day moving average of the present and previous 5 days. (vi) lag 06, the 7-day moving average of the present and previous 6 days.

GAM is a flexible regression analysis method that allows for the inclusion of non-linear relationships in the model and describes these relationships through non-parametric smooth functions (18). Firstly, we established a basic model that includes the long-term trend of time, the day of the week effect, and the holiday effect, as follows:


$$\begin{array}{l} Y_{t}\sim Poisson(u_{t})\\ Log(u_{t})=\beta \times s(X_{t},k)+s(time,df_{t}=7)+s(temperature,df_{t}\\ =6)+dow+\text{hol}+\alpha \end{array}$$

Among them, *Y*_t_ is the actual number of people in the hospital on the *t-*th day (following the Poisson distribution); μ_*t*_ = *E*[*Y*_*t*_]is the expected number of hospitalizations on the *t-*th day; β is the regression coefficient; *X*_t_ is the atmospheric pollutant element on the *t-*th day; *S* represents a non-parametric smoothing function, where *df*_t_ represents the degree of freedom of the long-term and seasonal trends of time;

In order to capture short-term fluctuations, the model incorporates holiday variables (hol) and week variables (dow); Holiday variables are simplified into binary categories, where hol = 1 represents holidays and hol = 0 represents non-holidays. The α in the model is the intercept term. In order to better capture the relationship and trend of changes between independent variables, cubic spline smoothing is used to map the discrete values of the independent variables to a continuous function, so that the model can better fit the data, i.e. *k* = 3; The degree of freedom of the annual non-parametric smoothing function for time is set to 7, a natural spline function with 6 degrees of freedom for daily mean temperature (19).

### 2.3 Statistical analysis

The relative risks (RR) with 95% confidence interval (CI) in thyroid diseases admissions associated with a 10.0 μg/m^3^ increase in daily concentration of NO_2_, SO_2_, and O_3_, and a 1.0 mg/m^3^ increase in daily concentration of CO were estimated, RR was calculated using the following formula (20):


$$\begin{array}{l} \begin{array}{l} RR=\exp\left[\beta *10\right]\times 100\%\\ 95\%\text{CI}=\exp[10*\left(\beta \pm 1.96SE\right]\times 100\% \end{array} \end{array}$$

where β is the regression coefficient from the GAM model, SE is the standard error.

Implementing stratified analysis to explore the impact of environmental pollutants on the risk of thyroid tumors in different subgroup variables included gender (male and female), age (<45, 45–65, and >65 years), and season (Spring, Summer, Autumn, and Winter).

By adjusting the degrees of freedom of the time smoothing function, we can monitor the sensitivity of the estimated effect values to changes in degrees of freedom and determine the stability of the model (21). R software (Version 3.2.3) was used to perform analysis, all the statistical tests were two-tailed and a *P* < 0.05 was considered statistically significant.

## 3 Result

### 3.1 Distribution characteristics of air pollutants and thyroid goiter

There were 37,630 hospital admissions for goiter in Luoyang from January 1, 2014 to July 30, 2022. Among them, there are 29,571 female (78.58%), 8,059 male (21.42%); 14,141 people (37.58%) from rural areas and 23,489 people (62.42%) from urban areas; The average age of the patient is 51 years old, 15,349 (40.79%) people are under 45 years old, 17,498 (46.5%) people are between 45 and 64 years old, and 4,783(12.71%) people are over 65 years old.

**Figure 2:**
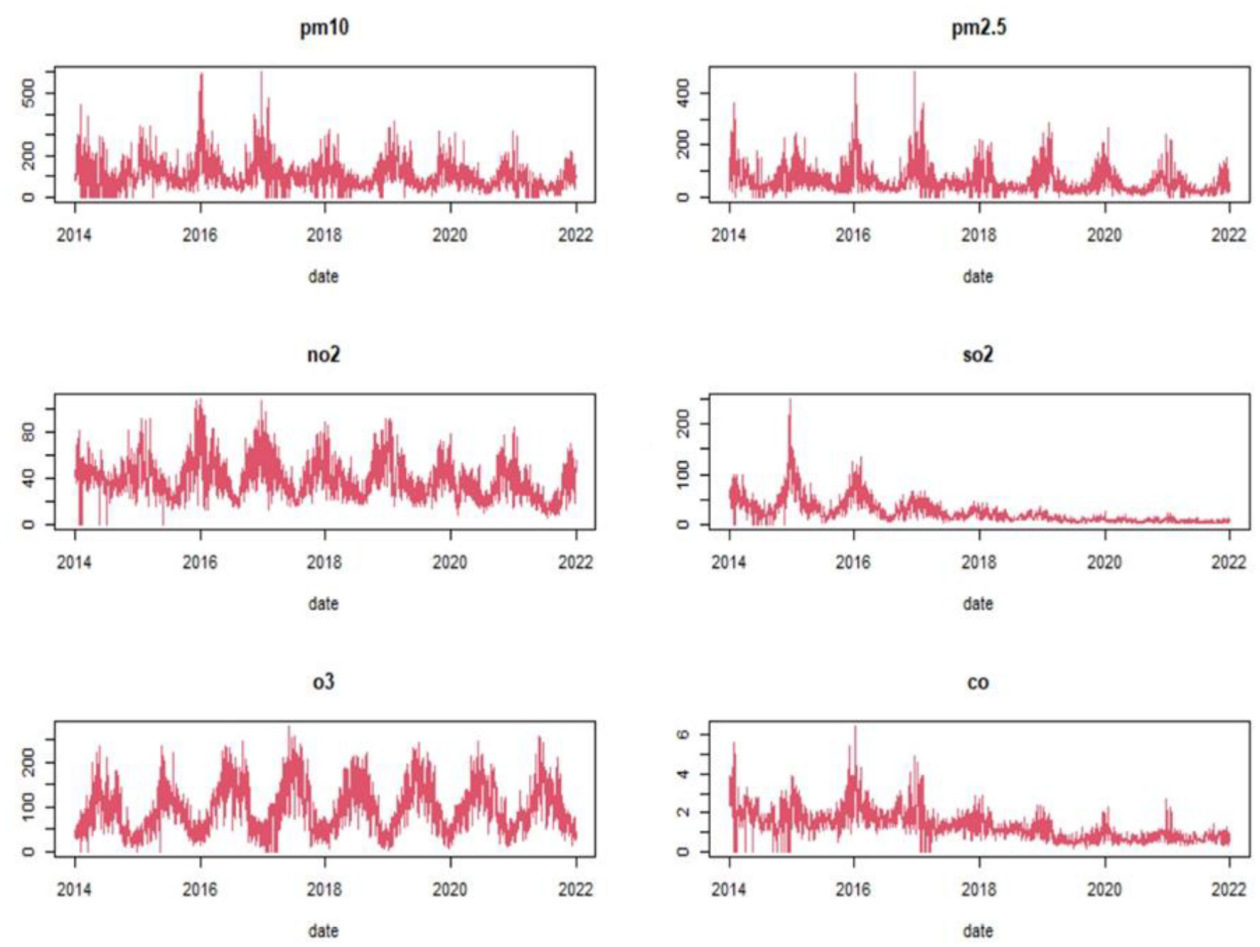
Time series changes in six pollutants concentration from 2014 to 2022.

Analysis of the air pollutants indicated the daily mean concentrations were 62.92 μg/m^3^ for PM_2.5_, 109.34 μg/m^3^ for PM_10_, 39.26 μg/m^3^ for NO_2_, 24.47 μg/m^3^ for SO_2_, 96.97 μg/m^3^ for O_3_, and 13.37 mg/m^3^ for CO (Table 1). The time series analysis of pollutant concentration suggests a decreasing and Seasonal trend in PM_2.5_, PM_10_, SO_2_, and CO from 2014 to 2022 (Figure 2).

**Table 1:** Descriptive statistics for the daily number of air pollution concentrations.

**Variables**	**Mean ±SD**	**Minimum**	***P*_25_**	**Median**	***P*_75_**	**Maximum**
PM_2.5_ (ug/m^3^)	62.92 ± 55.59	6	32	50	77	479
PM_10_ (ug/m^3^)	109.34 ± 56.25	8	66	99	132	599
O_3_ (ug/m^3^)	96.0.97 ± 31.83	5	55	89	132.25	279
NO_2_ (ug/m^3^)	39.0.26 ± 10.52	5	27	37	49	108
SO_2_ (ug/m^3^)	24.47 ± 14.41	2	9	16	32.25	249
CO (mg/m^3^)	13.37 ± 12.31	0.2	0.8	1.2	1.7	6.4

P_25_ 25th percentile, P_75_ 75th percentile, SD standard deviation.

### 3.2 Correlation analysis between various pollutants

**Figure 3:**
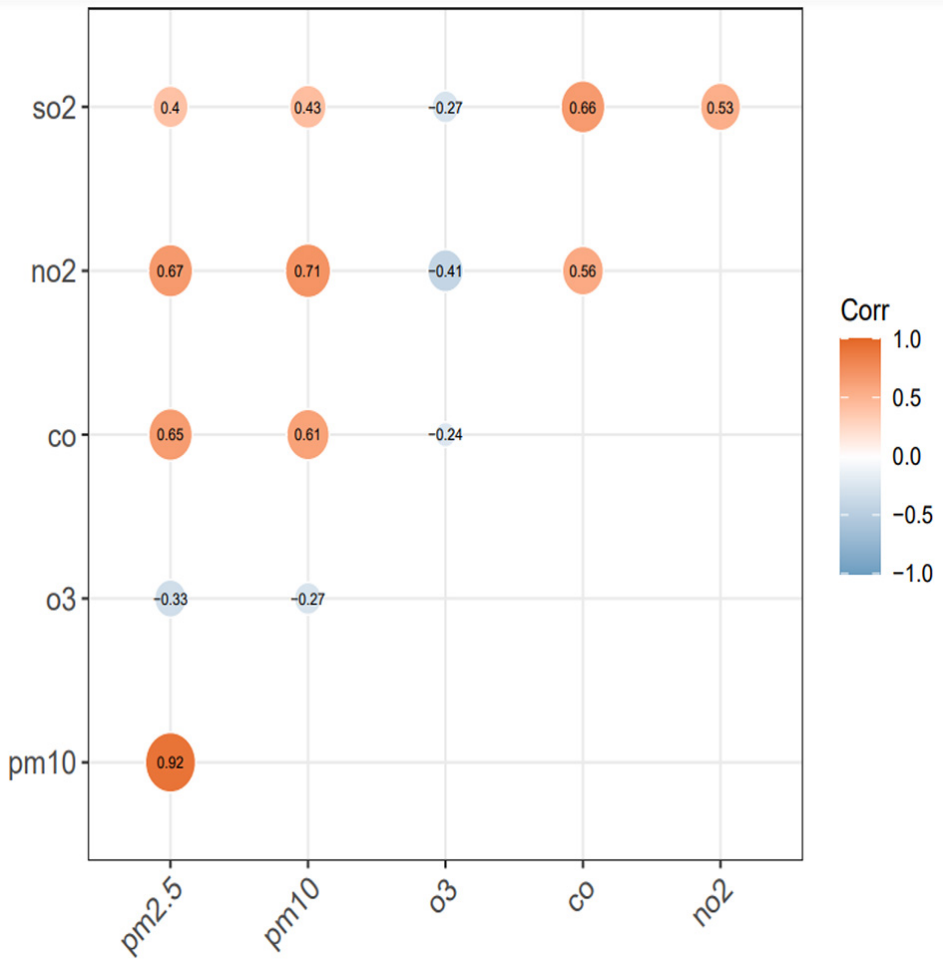
Spearman correlation coefficient matrix between six pollutants.

We calculated Spearman correlation coefficient (*r*) to examine the relationships of air pollutions (Figure 3). The results indicated that daily PM_2.5_ and PM_10_ concentrations had positive correlations with NO_2_ (PM2.5: *r* = 0.67, *P* < 0.001; PM_10_: *r* = 0.71, *P* < 0.001), SO_2_ (PM_2.5_: *r* = 0.4, *P* < 0.001; PM_10_: *r* = 0.43, *P* < 0.001), and CO (PM_2.5_: *r* = 0.65, *P* < 0.001; PM_10_: *r* = 0.61). O_3_ and other atmospheric pollutants show a negative correlation (*r* < 0, *P* < 0.001).

### 3.4 The lag effect of pollutants on the incidence of goiter

The impact of six pollutants had different lag effects on goiter (Table 2). The lag effect for PM_2.5_ and CO (lag1–3days) was significant and relatively longer for NO_2_ (lag0–3) and O_3_ (lag2–7). For PM_10_, the lag effect was significant only at lag1 and lag3. The accumulated lag effect for PM_2.5_ (lag01–06days), PM_10_ (lag01–03days), O_3_ (lag04–07), NO_2_ (lag00–07) and CO (lag01–05days) was significant and relatively longer. Besides, the lag effect for SO_2_ on goiter was not statistically significant.

**Table 2:** Delayed effect of single pollutant on goiter based on GAM model.

**Lag**	**PM**_**2.5**_		**PM**_**10**_		**O**_**3**_	
**Time (days)**	**RR**	**95%CI**	**RR**	**95%CI**	**RR**	**95%CI**
Lag0	1.0005	0.9967–1.0043	1.0009	0.9983–1.0035	0.9991	0.9950–1.0032
Lag1	**1.0064**	**1.0027–1.0101**	**1.0040**	**1.0014 1.0066**	0.9982	0.9941–1.0023
Lag2	**1.0057**	**1.0020–1.0094**	1.0012	0.9986–1.0039	**0.9952**	**0.9911–0.9993**
Lag3	**1.0064**	**1.0027–1.0101**	**1.0029**	**1.0003–1.0055**	**0.9954**	**0.9913–0.9995**
Lag4	1.0007	0.9970 −1.0045	0.9985	0.9959–1.0012	**0.9955**	**0.9915–0.9996**
Lag5	1.0020	0.9982–1.0059	0.9985	0.9959–1.0012	**0.9944**	**0.9904–0.9984**
Lag6	0.9979	0.9941–1.0018	0.9974	0.9948–1.0001	**0.9948**	**0.9907–0.9989**
Lag7	0.9950	0.9912–0.9988	0.9955	0.9929–0.9981	**0.9940**	**0.9899–0.9981**
**Lag**	**NO**_2_		**SO**_2_		**CO**	
**Time (days)**	**RR**	**95%CI**	**RR**	**95%CI**	**RR**	**95%CI**
Lag0	**1.0442**	**1.0320–1.0566**	1.0169	0.9998–1.0342	1.3971	0.9734–2.0052
Lag1	**1.0295**	**1.0176–1.0416**	1.0007	0.9834–1.0183	**1.5340**	**1.0726–2.1940**
Lag2	**1.0214**	**1.0096–1.0335**	0.9933	0.9761–1.0107	**1.5403**	**1.0758–2.2053**
Lag3	**1.0275**	**1.0155–1.0400**	0.9957	0.9785–1.0132	**1.6240**	**1.1347–2.3243**
Lag4	1.0113	0.9994–1.0232	0.9758	0.9588–0.9931	1.0623	0.7408–1.5233
Lag5	0.9919	0.9803–1.0037	0.9660	0.9491–0.9832	0.9666	0.6694–1.3955
Lag6	0.9737	0.9622–0.9854	0.9754	0.9587–0.9925	0.6858	0.4729–0.9947
Lag7	0.9679	0.9565–0.9795	0.9717	0.9549–0.9889	0.601	0.4187–0.8638
**Accumulated lag**	**PM**_2.5_		**PM**_10_		**O**_3_	
**Time (days)**	**RR**	**95%CI**	**RR**	**95%CI**	**RR**	**95%CI**
Lag00	1.0005	0.9967–1.0043	1.0009	0.9983–1.0035	0.9991	0.9950–1.0032
Lag01	**1.0044**	**1.0002–1.0087**	**1.0033**	**1.0003–1.0063**	0.9982	0.9935–1.0030
Lag02	**1.0066**	**1.0020–1.0113**	**1.0034**	**1.0001–1.0067**	0.9966	0.9908–1.0012
Lag03	**1.0088**	**1.0037–1.0139**	**1.0044**	**1.0008–1.0081**	0.9943	0.9887–1.0000
Lag04	**1.0086**	**1.0031–1.0141**	1.0035	0.9995–1.0075	**0.9928**	**0.9867–0.9988**
Lag05	**1.0092**	**1.0032–1.0152**	1.0028	0.9985–1.0071	**0.9907**	**0.9843–0.9972**
Lag06	**1.0083**	**1.0019–1.0148**	1.0017	0.9971–1.0063	**0.988**	**0.9821–0.9958**
Lag07	1.0062	0.9993–1.0130	0.9996	0.9947–1.0046	**0.9867**	**0.9795–0.99401**
**Accumulated lag**	**NO**_2_		**SO**_2_		**CO**	
**Time (days)**	**RR**	**95%CI**	**RR**	**95%CI**	**RR**	**95%CI**
Lag00	**1.0442**	**1.0320–1.0566**	1.0169	0.9998–1.0342	1.3971	0.9734–2.0052
Lag01	**1.0478**	**1.0339–1.0619**	1.011	0.9921–1.0320	**1.6272**	**1.0848–2.4408**
Lag02	**1.0503**	**1.0350–1.0659**	1.0061	0.9843–1.0284	**1.8719**	**1.1936–2.9355**
Lag03	**1.0585**	**1.0415- 1.0757**	1.0032	0.9800–1.0273	**2.1930**	**1.3405–3.5875**
Lag04	**1.0596**	**1.0413- 1.0783**	0.9926	0.9676–1.0182	**2.1193**	**1.2470- 3.6020**
Lag05	**1.0535**	**1.0339- 1.0735**	0.9797	0.9536–1.0066	**2.0254**	**1.1462–3.5791**
Lag06	**1.0410**	**1.0204–1.0620**	0.9720	0.9448–1.0000	1.7284	0.9408- 3.1751
Lag07	**1.0266**	**1.0051–1.0485**	0.9631	0.9348–0.9922	1.3960	0.7308- 2.6666

Bold parts indicate statistical significance (*P* < 0.05).

Specifically, a 10μg/m^3^ increase in PM_2.5_, PM_10_, NO_2_ and a 1μg/m^3^ increase in CO was associated with 0.92% (RR: 1.0092; 95%CI: 1.0032–1.015),0.44% (RR:1.0044; 95% CI: 1.0008–1.0081), 5.96% (RR: 1.0596; 95% CI: 1.0413, 1.0783) and 62.4% (RR: 1.624; 95% CI: 1.1347, 2.3243) increased risk of goiter on the optimal lag day.

### 3.5 Response-relationship between pollutants and the number of patients with goiter

**Figure 4:**
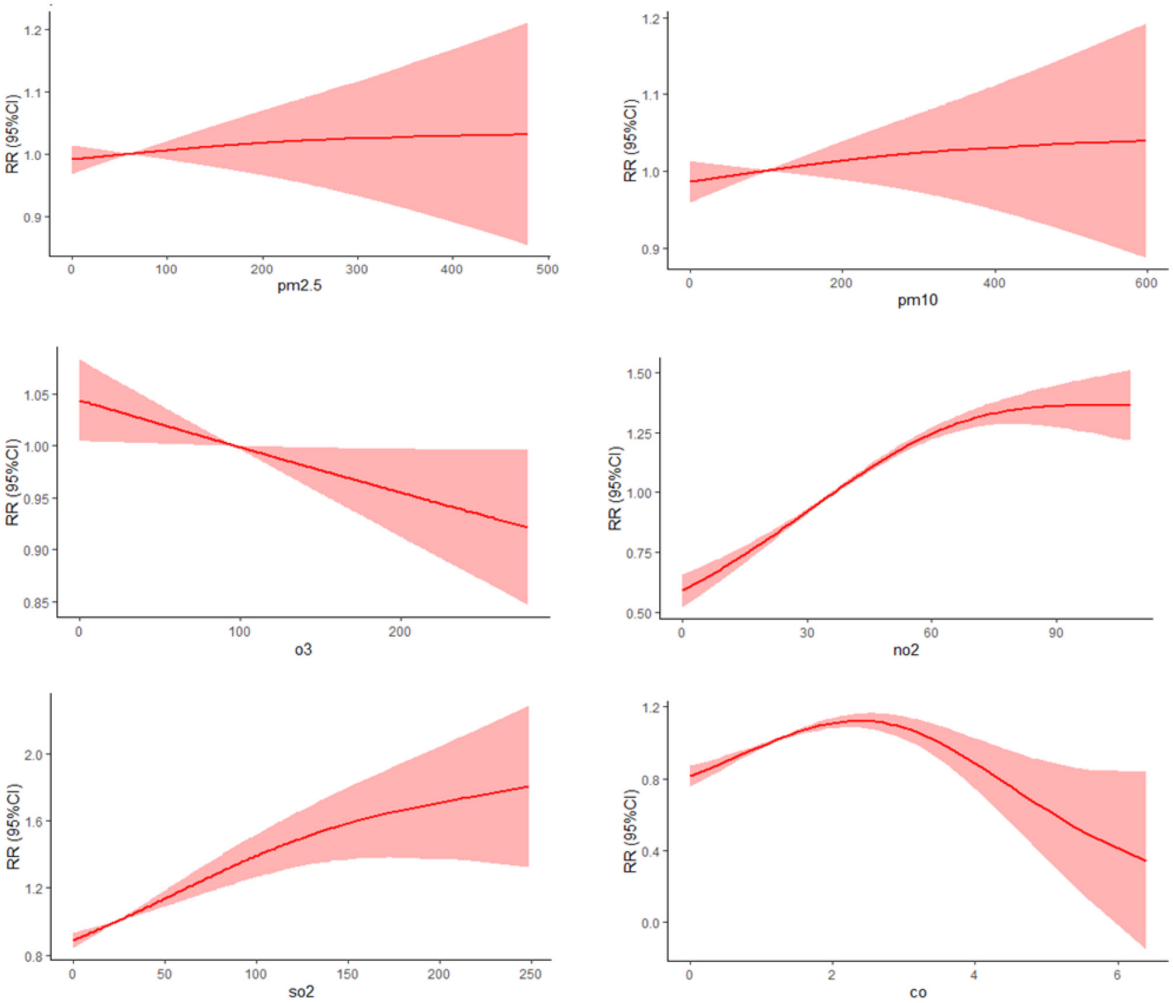
Dose relationship curve between air pollutants and goiter.

The dose-response relationship between pollutants concentration and goiter were shown in Figure 4. As the concentration of PM_2.5_ and PM_10_ increases, the risk of goiter showed a near linear increase trend, and an exponential increase trend with NO_2_ and SO_2_ increase. O_3_ exhibits a protective effect against goiter, and as O_3_ concentration increases, the risk of goiter decreases continuously. As the concentration of CO increases, the risk of disease shows a trend of first increasing and then slowly decreasing.

### 3.6 Stratified analyses by gender, age, and season

**Figure 5:**
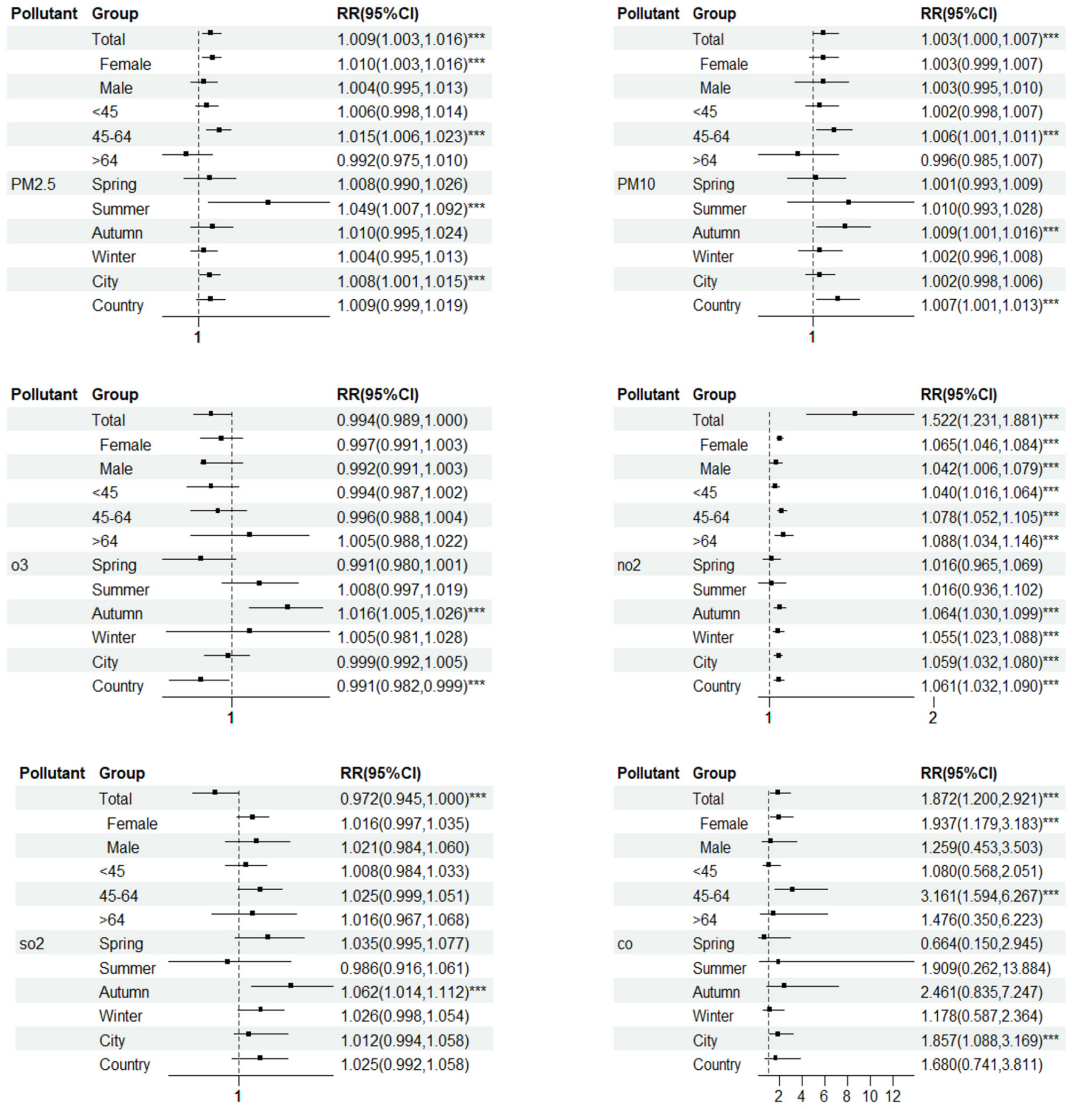
The health effects of pollutants on different population groups. ***Represents statistically significant (*P* < 0.05).

In gender stratification, PM_2.5_ and CO have statistical significance for the female population, while NO_2_ has statistical significance for both males and females. Women are more sensitive to NO_2_ than men. When NO_2_ concentration increases by 10 μg/m3, the RR of goiter in female and male populations are 1.065(1.046,1.084) and 1.042(1.006,1.079), respectively; When the concentration of PM_2.5_ and CO increases by 10 μg/m^3^ and 1 mg/m^3^ separately, the RR of goiter in the female population are 1.01(1.003,1.016) and 1.937 (1.179,3.183) (Figure 5).

In different age groups, PM_2.5_, PM_10_, and CO have significant effects on the population aged 45–65. When the concentration of pollutants increases by 10 μg/m^3^ (CO increases by 1mg/m^3^), the RR of goiter increased by 1.48%, 0.61%, and 3.1609 times, respectively; NO_2_ has statistical significance in all age groups. When NO_2_ concentration increases by 10 μg/m^3^, the RR of goiter in <45 years old, 45–64 years old, and >65 years old age groups increased by 3.96%, 7.79%, and 8.8%, respectively (Figure 5).

PM_2.5_ had a significant effect on goiter in Summer. When the pollutant concentration increases by 10 μ g/m^3^, the RR of goiter increased by 4.87%; However, PM_10_, NO_2_, and SO_2_ have a more significant impact on goiter in autumn. When the pollutant concentration increases by 10 μ g/m^3^, the RR of goiter increased by 0.87%, 6.38%, and 12.44%, respectively; In winter, NO_2_ has a more significant impact on goiter. When the concentration of pollutants increases by 10 μg/m^3^, the RR of goiter increased by 5.48% (Figure 5).

### 3.7 Dual pollutant analysis

There is an interactive effect between pollutants on diseases. Except for O_3_, other pollutants have a certain synergistic effect on thyroid goiter. Adding PM_10_ to PM_2.5_ has the significant impact on goiter, with a 1.0111% (95% CI: 1.0051, 1.0171) risk for goiter; Adding NO_2_ to PM_2.5_ with a 1.0701% (95% CI: 1.0512, 1.0894) risk for goiter; Adding PM_2.5_, PM_10_, O_3_, NO_2_, SO_2_ separately to CO may increase the risk of thyroid goiter by 2.5746 times(RR:2.5746, 95% CI: 1.4894–4.4505), 2.34 times(RR:2.34, 95% CI: 1.3845–3.955), 2.1893 times(RR:2.1893, 95% CI: 1.3383–3.5815),1.6727 times (RR:1.6727, 95% CI: 1.0053–2.7832) and 2.2812 times (RR:2.2812, 95% CI: 1.3908–3.7416). However, O_3_ exhibited antagonistic effects against other pollutants.

### 3.8 Model fitting results and verification

The GAM model was used to fit each pollutant data separately, and all smoothing terms reached significance at the *p* < 0.05 level (Table 3). After adjusting the interference factor temperature in the model, the relative risk has decreased slightly, but it is still statistically significant. The adjusted *R*^2^ values of each model in Table 3 are around 80%, indicating a good fitting effect of the GAM model.

**Table 3:** Adjusted RR for the association of air pollutants and thyroid goiter.

**Variables**	**Lag**	**Unadjusted RR (95%CI)**	**Adjustment for temperature**	**Adjusted *R*^2^**	***p*-value**
PM_2.5_	Lag3	1.0064 (1.0027–1.0110)	1.0047 (1.002–1.0095)	0.839	<0.01
PM_10_	Lag1	1.0040 (1.0014–1.0066)	1.0022 (1.0014–1.0039)	0.833	<0.01
O_3_	Lag4	0.9955 (0.9915–0.9996)	0.9913 (0.9915–0.9996)	0.858	<0.01
CO	Lag3	1.6240 (1.1347–2.3243)	1.3971 (1.0954–1.9052)	0.743	0.025
NO_2_	Lag0	1.0442 (1.032–1.0566)	1.0214 (1.0155–1.0464)	0.812	<0.01
SO_2_	lag0	1.0169 (0.9998–1.0342)	0.9957 (0.9598–1.0183)	0.820	<0.01

### 3.9 Sensitivity analysis

The degrees of freedom for selecting time are 5, 6, 7, 8, and 9, respectively. The effects of PM_2.5_, PM_10_, NO_2_, and CO on the risk of goiter are statistically significant, and according to the increase in degrees of freedom, it can be seen that there is little change in the RR with 95% confidence interval, indicating that the model is relatively stable (Table 4).

**Table 4:** The effect of increasing pollutants concentration by 10 μg/m^3^ (CO increase by 1 mg/m^3^) at different degrees of freedom on goiter.

**Degree of freedom**	**PM**_**2.5**_		**PM**_**10**_		**O**_**3**_	
	**RR**	**95%CI**	**RR**	**95%CI**	**RR**	**95%CI**
d*f* = 5	1.0085	1.0031–1.0139	1.0034	1.0001–1.0066	0.9962	0.9911–1.0012
d*f* = 6	1.0092	1.0038–1.0147	1.0034	1.0001–1.0067	0.9951	0.9900–1.0003
d*f* = 7	1.0087	1.0032–1.0143	1.0034	1.0001–1.0067	0.9959	0.9907–1.0011
d*f* = 8	1.0112	1.0056–1.0168	1.0050	1.0016–1.0084	0.9952	0.9900–1.0004
d*f* = 9	1.0104	1.0048–1.0161	1.0046	1.0012–1.0080	0.9950	0.9897–1.0003
	**NO**_2_		**SO**_2_		**CO**	
	**RR**	**95%CI**	**RR**	**95%CI**	**RR**	**95%CI**
d*f* = 5	1.0604	1.0438–1.0773	0.9883	0.9640–1.0133	1.7346	1.1145–2.6996
d*f* = 6	1.0612	1.0443–1.0784	0.9810	0.9559–1.0068	1.8720	1.1998–2.9208
d*f* = 7	1.0589	1.0419–1.0762	0.9800	0.9540–1.0067	1.8665	1.1899–2.9282
d*f* = 8	1.0616	1.0443–1.0792	0.9790	0.9518–1.0071	1.8604	1.1801–2.9329
d*f* = 9	1.0613	1.0437–1.0792	0.9747	0.9471 1.0031	1.6879	1.0674–2.6692

## 4 Discussion

The present study suggests that long-term exposure to air pollutions (PM_2.5_, PM_10_, NO_2_, CO) may be associated with an increased risk of goiter diseases based on a 9 years of time series data in Luoyang.

In our study, we found that females patients with goiter are predominant, 78.58% female, 21.42% male, which goes with study in Diwaniyah Teaching hospital of Iraq by Adel Mosa et al. in which 74.3% of patients was females (1). In our study, the mean age of patients was 51 years old, this is more than that reported by Mishra (48 year) (22). The commonest ages at presentation were (45–64 years), this is almost consistent with previous studies (23, 24). Most patients (62.42%) come from urban areas, which may be due to more severe air pollution in cities.

In line with several previous studies (25, 26), we found that PM_2.5_ had a greater impact on thyroid diseases than PM_10_ at all lag structures. Compared to PM_10_, PM_2.5_ adsorbs toxic substances and heavy metals more readily due to its larger relative surface area, it remains suspended in the atmosphere for longer periods, and it enters the skin and even the bloodstream more easily (27). However, the impact of CO and NO_2_ on the goiter exceeds that of PM_2.5_, NO_2_, and CO are toxic gases that are reported to cause harm to the human respiratory, cardiovascular, and nervous systems. We found a significant correlation between NO_2_, CO and thyroid goiter. A 10μg/m^3^ increase in NO_2_ concentration and a 1μg/m^3^ increase in CO was associated with 5.96% and 62.4% increased risk of goiter. CO can form carboxyhemoglobin with free thyroxine in the body, and this hemoglobin can serve as a target for cancer cells to some extent, causing hyperthyroidism and further inducing cancer (28). The study also found that long term inhalation of high concentrations of NO_2_ may increase the risk of thyroid cancer (29).

In gender stratification, PM_2.5_, NO_2_, and CO have a more significant impact on the female population. Although we haven't fully understood the mechanism, numerous pieces of evidence indicate that air pollution is more harmful to women than men, multiple studies also support this conclusion (30, 31). According to the WHO, in 2012, over 60% of premature deaths caused by indoor air pollution were women and children. Our age-stratified analysis found that PM_2.5_, PM_10_, NO_2_ and CO have significant effects on the population aged 45–65, this may be due to the fact that this age group has the highest number of patients, and multiple studies have reported that air pollutants cause the greatest harm to middle-aged and older people (29, 32). We found a significant association between pollutions and thyroid goiter in autumn, but not in warm seasons, consistent with previous studies on PM and respiratory diseases (27). A recent study also has found significant seasonal variations in thyroid stimulating hormone (TSH) and thyroid hormone levels (T3, FT3, T4, FT4) (33).

Concentrations exposure-response relationships showed a near linear increased trend between PM and goiter, and an exponential increased trend with NO_2_ and SO_2_ increase; China is one of the most polluted countries in the world due to the rapid industrialization and urbanization. However, as the concentration of CO increases, the risk of disease shows a trend of first increasing and then slowly decreasing. Chen et al. (34) also reported the same result in their study on the impact of CO on the incidence of conjunctivitis, with a weaker effect at higher concentrations. This non-linear relationship may be because people avoid spending time outside or wear a dust mask when outside when the air is heavily polluted (25).

Our two-pollutant model indicated that the association between pollutions and thyroid diseases remained positive and the risk increased, but not significant after adjusting for O_3_. The addition of NO_2_ to other pollutants showed statistical significance within 1–7 days. The addition of NO_2_ to PM_2.5_, PM_10_, and CO all reached their maximum effect values on the third day, while the addition of SO_2_ reached its maximum effect on the 4th day. The addition of CO to PM_2.5_, PM_10_, O_3_, and SO_2_ has statistical significance from 1 to 5 days, and reaches the maximum effect value on the third day. These results indicate that there is a synergistic effect between PM_2.5_, PM_10_, NO_2_, and CO, while O_3_ has an antagonistic effect with other pollutants. Fervers et al. (35) also reported similar results regarding air pollutants and breast cancer risk.

## 5 Potential mechanism

There are several possible reasons to explain the impact of air pollutants on thyroid goiter. Hypothyroidism or chronic inflammation resulting in decreased thyroid function, elevated TSH levels in patients, and further growth of the thyroid gland after TSH elevation, resulting in gradual increase in thyroid volume, is currently a common thyroid goiter (36); According to reports, air pollution has a significant impact on hypothyroidism (37); Inflammatory lesions can cause bleeding, increased fluid in the thyroid gland, or other conditions that lead to an increase in thyroid volume; Thyroid tumors, including benign and malignant tumors, can lead to goiter. It's reported by Al-Rekabi and Habban (1), thyroid tumor rate was 21.6% from patients with goiter and Eusebio Chiefari reported this proportion was 12.5% in Italy (38). Several studies also found that air pollution increases the risk of thyroid tumors (39, 40).

## 6 Limitations

Despite providing direct evidence for the association between air pollution and thyroid goiter, this study has several limitations. Firstly, this study was conducted based on existing air quality monitoring data of Luoyang and goiter records in hospitals, there may be a data gap, which may impact the results and lead to either underestimation or overestimation of air pollutants exposure levels. Secondly, this study did not fully consider other environmental factors that may affect goiter, such as humidity, atmospheric pressure, etc., which may interact with air pollutants and influenza the incidence of goiter. Further research is needed to explore the association of climate conditions with thyroid diseases.

## 7 Conclusion

The main results of this study suggested that long-term exposure to air pollutants (PM_2.5_, PM_10_, NO_2_, and CO) may be associated with an increased risk of thyroid diseases. These associations were stronger for people more than 45 years old and during the autumn, especially for women. These findings have important implications for policymakers to take concrete actions to reduce atmospheric pollutions concentrations and protect the environment.

## Data Availability

The raw data supporting the conclusions of this article will be made available by the authors, without undue reservation.
